# Catatonia and melancholia interface: exploring a new paradigm for evaluation and treatment. A case series and literature review

**DOI:** 10.3389/fpsyt.2024.1372136

**Published:** 2024-03-20

**Authors:** Yassir Mahgoub, Aum Pathare, Dallas Hamlin, Hassaan Gomaa, Sean Nutting, Charles Mormando, Andrew Francis

**Affiliations:** ^1^Department of Psychiatry and Behavioral Health, College of Medicine, The Pennsylvania State University, Hershey, PA, United States; ^2^Department of Psychiatry, Albert Einstein Medical Center - Jefferson Health, Philadelphia, PA, United States

**Keywords:** melancholia, catatonia, depression, bipolar disorder, lorazepam

## Abstract

**Background:**

Catatonia has been increasingly associated with mood disorders and is recognized as a specifier in the DSM-5 and DSM-5-TR. The DSM-5-TR recognizes melancholia as a specifier for depressive episodes in major depressive disorder and bipolar disorder. It is characterized by severe anhedonia, lack of reactivity, excessive or delusional guilt, and significant vegetative symptoms. As the conceptualization of melancholia expanded beyond its mood components to include psychomotor disturbances, its overlap with psychomotor symptoms or catatonia becomes evident. This overlap was also described in Kahlbaum’s original literature, where he describes the transition between states of melancholia, mania, and catatonia.

**Method:**

Case summary of six patients with major depressive disorder or depressed phase of bipolar disorder who were admitted for severe depression, anhedonia, intense anxiety, psychomotor agitation or retardation, indecisiveness, perseveration, and vegetative symptoms such as poor sleep, appetite, and significant weight loss.

**Results:**

All patients demonstrated rapid and complete resolution of their mood and psychomotor symptoms, indecisiveness, perseveration, as well as psychosis shortly after administration of lorazepam, with recurrence of the above symptoms upon lorazepam discontinuation and resolution upon resumption, in an on-and-off manner.

**Conclusion:**

The present study argues for a closer relationship between melancholia and catatonia based on our case series, historical review, overlap in phenomenology, and response to treatment. We propose provisional [Mahgoub] criteria for patients with severe depression and melancholia. The role of GABA agonists, such as lorazepam, can be explored as an option for patients with treatment-resistant depression who meet these criteria for melancholia.

**Limitations:**

Absence of a standardized, systematic assessment tool and a small sample size.

## 1 Introduction

Despite the broad association of melancholia with severe episodes in mood disorders and catatonia as a predominant psychomotor disorder, a significant body of literature suggests a comprehensive overlap between them that includes prominent affective, cognitive, and psychomotor manifestations (1, 2).

Kahlbaum, who coined the term catatonia, noted that melancholia could progress into catatonia and later described how catatonia could emerge during the transition between melancholia and mania (3). He described a variant of melancholia, referred to as “*melancholia attonita” or “astonished melancholia,”* that presents with prominent motor and cognitive manifestations, such as stupor, cognitive decline, and the frozen motor manifestation (4). He highlighted that abnormal motility marks the difference between classic melancholia and melancholia attonita. Furthermore, he explained that when these patients develop astonishment, they cross the border to melancholia attonita and named it the “Kahlbaum border.” He gradually added more signs and called the condition catatonia, which he described as a brain disease with a cyclic alternating course, in which the longitudinal course of mental symptoms is consecutively melancholy, mania, stupor, confusion, and eventually dementia (3).

Kahlbaum’s initial observations about catatonia were later recognized as a foundation of the diagnostic criteria for the syndrome. He also made an essential link between melancholia and catatonia, suggesting an overlap in symptoms or phases of illness (4).

### 1.1 The dichotomy between melancholia and catatonia

Later writers, especially Kraepelin and Bleuler, associated catatonia exclusively with motor symptoms of schizophrenia and neglected its affective manifestations (5, 6). This resultant separation led to catatonia being recognized as a psychomotor syndrome and as a subtype of schizophrenia, while melancholia remained linked to mood disorders (7–11).

Due to the significant correlation between catatonia and mood disorders (12), catatonia was later recognized as co-occurring with mood disorders, schizophrenia, and other medical conditions in the DSM-5 (11). However, symptoms of catatonia were dominated by motor and behavioral presentations. According to the current DSM-5-TR criteria, the diagnosis of catatonia requires the presence of 3 out of the 12 following criteria: catalepsy, waxy flexibility, stupor, agitation, mutism, negativism, posturing, mannerisms, stereotypies, grimacing, echolalia, and echopraxia (13). **Table 1** describes the DSM-5-TR criteria for catatonia.

**Table 1:** DSM-5-TR criteria for catatonia: The patient must meet three out of the following twelve symptoms of catatonia (13).

Symptoms	Definition
1. Catalepsy	Passive induction of postures held against the gravity
2. Waxy flexibility	Slight and even resistance to repositioning by the examiner
3. Stupor	No psychomotor activity, no reactivity to the environment
4. Agitation	Not influenced by external stimuli
5. Mutism	No or minimal verbal response- not applicable in case of established aphasia
6. Negativism	Opposing or not responding to external stimuli or instructions
7. Posturing	Spontaneous and active maintenance of posture against gravity
8. Mannerism	Odd caricature of ordinary actions
9. Stereotypies	Repetitive, frequent, non-goal directed movements
10. Grimacing	Maintenance of odd facial expression
11. Echolalia	Repeating the words spoken by the examiner
12. Echopraxia	Mimicking of movements made by the examiner

The representation of motor and behavioral manifestation also dominated all the current seven clinical scales utilized for recognition and monitoring of catatonia, such as the Bush Francis Catatonia Rating Scale (BFCRS) (14), Modified Rogers Scale (MRS-C) (15), Rogers Catatonia Scale (RCS) (16), Kanner Scale (Kanner) (17), Brauning Catatonia Rating Scale (BCRS) (18), and the Pediatric Catatonia Rating Scale (PCRS) (19). However, the Northoff Catatonia Rating Scale (NCRS) was the exception for recognizing affective symptoms, such as anxiety, flat affect, affective latency, compulsive emotions, and emotional lability (2).

With the recognition of melancholia as a mood presentation, Kraepelin coined the term manic-depressive insanity to describe what is currently known as bipolar disorder and major depressive disorder. He systematically described the mixed affective states, noting excitement or depression of three different domains of psychiatric life: the intellect, mood, and psychomotor activity or volition (20). Bonhoeffer categorized depression into “endogenous depression” and “symptomatic depression,” suggesting a biological role in the first or being linked to somatic illness as opposed to “psychogenic depression,” which was attributed to life events (21).

Koukopoulos and Koukopoulos studied agitated and mixed depression, wherein mood, affective, and psychomotor symptoms did not necessarily share the same valence (22). They described a form of mixed depression, characterized by marked psychomotor or inner excitation along with severe depression, where patients tend to have physical aggression, significant mood lability, rage, anger, and inner tension as opposed to the classic melancholic states that are predominated in his view by psychomotor retardation, anhedonia, and anergia (22). Several authors followed his lead and reported a severe course of illness, the presence of psychotic symptoms, frequent hospitalizations, high risk of suicide, and non-response or even worsening with antidepressants (23, 24). Similar to melancholic depression, Kouropoulos et al. suggested that these patients tend to respond favorably to electro-convulsive treatment (ECT) treatment in addition to antipsychotics and benzodiazepines (25).

DSM-III changed melancholia to a diagnostic specifier under a major depressive disorder or bipolar disorder, where its listed criteria overlapped with major depression (7). In DSM-III-R and subsequently, DSM-IV, DSM-IV-TR, DSM-5, and DSM-5-TR, melancholic features are considered specifiers and defined by symptom aggregation alone with no particular relevance to psychomotor or vegetative symptoms (8–11, 13). Melancholia as a specifier can be applied to the current or most recent major depressive episode in major depressive disorder or bipolar disorder (**Table 2**).

**Table 2:** Diagnostic criteria for a major depressive episode with melancholia according to the DSM-3-R and both DSM-4 and DSM-5 and DSM-5-TR (7–11, 13).

DSM-3-R	DSM-4, DSM-4-TR, DSM-5 and DSM-5-TR
At least 5 of the following:	Either no. 1 or 2 and the last 3 of no 3-8:
1. Loss of pleasure in all or almost all activities	1. Loss of pleasure in all or almost all activities
2. Lack of reactivity to usually pleasurable stimuli.	2. Lack of reactivity to usually pleasurable stimuli.
3. Depression is usually worse in the morning	3. Depression is usually worse in the morning
4. Early morning awakening	4. Early morning awakening
5. Marked psychomotor retardation or agitation.	5. Marked psychomotor retardation or agitation.
6. Significant anorexia or weight loss	6. Significant anorexia or weight loss
7. No significant personality disturbances before a first major depressive episode.	7. Excessive or inappropriate guilt
8. One or more major previous depressive episodes followed by complete or nearly complete recovery	8. Distinct quality of depressed mood
9. Previous good response to previous and adequate somatic antidepressant treatment.	

Fink et al. criticized the first two DSM-IV-TR criteria for mood reactivity in melancholic depression for not being specific enough. He also noted that psychomotor and vegetative changes are not mandatory for diagnosis (26). Both Fink et al. and Parker & McCraw argued that the DSM constructs do not predict response to treatment, particularly ECT (26, 27). Rush and Weissenburger found that psychomotor changes are the main distinguishing features of melancholia, which can also predict response to ECT (28). Hickie et al. emphasized that vegetative and psychomotor symptoms are core features of the disorder and predict treatment response with ECT (29).

The current DSM-5-TR recognizes melancholia as an episode specifier for major depressive disorder and bipolar disorder, characterized by severe anhedonia, lack of reactivity, and excessive or delusional guilt with significant vegetative symptoms (13).

### 1.2 Recent recognition of the overlap between catatonia and melancholia

As stated above, Northoff et al. described various affective manifestations of catatonia and highlighted that it is a syndrome that has psychomotor, affective, and behavioral manifestations (2). On the other hand, Fink and Taylor suggested that melancholia is a syndrome that has three major symptom clusters that involve apprehension, anhedonia, and overwhelming gloom (30). In addition, Fink’s conceptualization included motor and cognitive symptoms involving either psychomotor slowness or even catatonia, agitation, restlessness, and preservative ruminations and actions. They included prominent disturbances in body functions or vegetative signs, such as sleep, appetite, and weight; thoughts of death, suicide, and delusions are common without listing them as part of the criteria and note that Hypothalamic-Pituitary-Adrenal (HPA) test abnormality Dexamethasone Suppression Test (DST), Corticotropin-Releasing Hormone (CRH) or high nighttime cortisol levels, decreased sleep latency, or other sleep abnormalities are common biological markers. They postulate that a response to ECT is even more pronounced in psychotic depression than non-psychotic melancholia (30, 31). Such a definition not only mentioned catatonia as a presentation seen among patients with melancholia but also suggested various motor and cognitive clusters that resemble features of catatonia.

In summary, the historical concept of melancholia represented a severe variant of depression with mood symptoms, abnormal thought processes and content, and psychomotor agitation or retardation. The recent DSM melancholia diagnostic construct has been criticized for its lack of specificity or neglect of its psychomotor features, which have been proven to be predictors for response to treatment.

## 2 Methods

Case summary of six patients with major depressive disorder or bipolar disorder who were treated in an inpatient facility for severe depressive episodes with significant psychomotor agitation or retardation. These patients were admitted to an academic inpatient psychiatric hospital in Central Pennsylvania between 2019 and 2022 and were under treatment by the research team. The identified subjects met the following criteria:

1. Primary admission diagnosis of major depressive disorder or bipolar disorder.
2. Admission with a depressive episode.
3. Presence of all the following symptoms: severe depressed mood, anhedonia, indecisiveness, perseveration, psychomotor agitation or retardation, and severe vegetative symptoms.

Patients with melancholia were followed clinically. For case 1, the Bush Francis Catatonia Rating Scale (BFCRS) was used to assess the progress of catatonia. The research team treated these subjects and was monitored clinically during their hospitalization.

All subjects provided written consent for publication after their treatment on their day of discharge. The Penn State IRB department provided IRB exemption for submission ID: STUDY00021722.

## 3 Results

The following case series summarizes the course of patients with major depressive disorder or bipolar disorder who were admitted for severe depression, significant psychomotor agitation or retardation, prominent vegetative symptoms, perseveration, and indecisiveness. It is noteworthy to mention that 5/6 had delusions during their course of illness. Most of these patients had inadequate or worsening symptoms with the use of antidepressants. However, these patients showed rapid and complete resolution of the above symptoms using lorazepam or other GABA agonists, like zolpidem. There was a recurrence of symptoms upon lorazepam discontinuation and resolution of symptoms with resumption of lorazepam in a clear on-and-off manner. Such response to lorazepam is known for catatonia but is not commonly recognized for mood or psychotic symptoms. Thus, these cases highlight the overlap between melancholia and catatonia, provide guidance for criteria of symptoms that might better represent melancholia and its overlap with catatonia, and potentially predict a positive response to treatment with lorazepam or other GABA agonists.

### 3.1 Case 1

A 61-year-old female with no significant medical history and a past psychiatric history of recurrent major depressive disorder with psychotic features was hospitalized following an interrupted suicidal attempt via stabbing in the context of worsening depression over the prior four months. Before hospitalization, she had been maintained on olanzapine 10 mg daily, sertraline 100 mg daily, and alprazolam 0.5 mg daily as needed for anxiety, with good medication adherence.

She presented with persistent low mood, anhedonia, increased psychomotor agitation, restlessness, poor sleep, and poor appetite. Additionally, she had occasional mutism, staring, motor and cognitive indecisiveness, negativism, and crying spells. She had marked perseveration and delusional content about causing her husband’s malignancy through her remote history of sexually transmitted disease. She had excessive fear of losing her house and becoming homeless. Her past psychiatric history was notable for similar episodes of depression characterized by significant psychomotor retardation, poor oral intake with failure to thrive, and mutism. She was diagnosed with recurrent major depression with psychotic features in addition to catatonia. Her initial BFCRS score was 12.

Following her hospitalization, she was started on olanzapine 2.5 mg daily, escitalopram 10 mg daily, and lorazepam 1 mg twice daily for catatonia. Most of the catatonic symptoms, apart from mild excitement and thought perseveration, resolved within one day following the above changes. However, her mood continued to remain low, and she continued to express delusions about her husband’s health. On day 3, lorazepam was increased to 2.5 mg twice daily, with subsequent resolution of catatonia. Interestingly, her mood and psychotic symptoms also improved following this change.

Due to concerns about the habit-forming nature of lorazepam, she requested that we taper down her off. Hence, lorazepam was gradually reduced and discontinued over five days. On day ten, symptoms of depression, poor oral intake, psychomotor agitation, and delusions about her husband dying from cancer and becoming homeless reemerged. Hence, lorazepam was resumed at 1.5 mg twice daily, and these reemergent symptoms resolved within 48 hours. ECT was pursued based on the patient’s request, and she received six treatments of bilateral ECT with benefits. Lorazepam was reduced gradually and stopped, and she was discharged with maintenance ECT as an outpatient, with no symptom recurrence for one year.

### 3.2 Case 2

A 60-year-old male with a history of recurrent major depressive disorder and no other medical problems was admitted to the inpatient unit for worsening depressive symptoms and suicidal ideation in the context of psychosocial stressors. His depressive symptoms had been worsening for three months before presentation as he experienced severely depressed mood, decreased appetite and a 20-pound weight loss, decreased energy, feelings of guilt, poor sleep, and suicidal ideations for over the two months before this hospitalization. He had been on escitalopram 20 mg daily for two months before this admission. He noted worsening depression despite medication adherence and the efficacy of escitalopram in successfully treating his depression years ago.

Escitalopram was continued at 20 mg daily, and mirtazapine 15 mg daily was added for augmentation. Subsequently, he demonstrated worsened depressive symptoms with prominent guilt, hopelessness, anxiety, and anhedonia. He showed substantial psychomotor retardation and became isolated to his room. His appetite decreased, and he stopped showering with worsening self-hygiene.

Due to the worsening of symptoms and apparent lack of efficacy of escitalopram, it was cross-titrated with fluoxetine 20 mg daily on day three. However, his depressive symptoms worsened further, and ECT was considered. He declined ECT and demonstrated significant indecisiveness and inability to consent to this procedure. He appeared to be internally preoccupied, endorsed both nihilistic and paranoid delusions, and demonstrated perseveration. Olanzapine 2.5 mg daily and lorazepam 0.5 mg twice daily were added to treat agitation on day five. Before and after receiving olanzapine and within a few hours after receiving the first dose of lorazepam, his mood showed significant improvement. He was noted to be more interactive, less anxious, and more energetic. Delusions and perseveration improved; hence, lorazepam was discontinued on day six. However, the prior depressive and psychotic symptoms reemerged on day eight. Mirtazapine was discontinued, and fluoxetine increased to 40 mg daily on day 8, which resulted in worsening anxiety and mood. Hence, lorazepam 1 mg twice daily was added. This resulted in a notable improvement in mood, isolation, energy, anhedonia, delusions, and psychomotor retardation within the same day. As his symptoms improved, lorazepam was reduced gradually and discontinued by day 13. However, depressive symptoms and psychotic symptoms recurred on day 15. No notable benzodiazepine withdrawal symptoms were associated with the reduction and discontinuation of lorazepam, and the patient was still maintained on olanzapine during the emergence of the above symptoms. Hence, lorazepam was restarted at 0.5 mg twice daily, and his mood again improved with the resolution of psychotic symptoms. He was discharged on fluoxetine 40 mg daily, olanzapine 2.5 mg daily, and lorazepam 0.5 mg twice daily. Six months after discharge, he had another severe depressive episode and required rehospitalization. Lorazepam was used for treatment, and it helped improve his mood and psychomotor symptoms. ECT was initiated, and his symptoms resolved entirely. Lorazepam was subsequently discontinued, and continued maintenance of ECT in the outpatient setting with good compliance for four months following discharge.

### 3.3 Case 3

A 54-year-old male with bipolar I disorder and no relevant medical history, with multiple psychiatric admissions due to manic and depressive episodes, presented to the emergency department with extreme irritability, paranoia, and agitation. He also had a history of an alcohol use disorder, with his last drink being 35 days before admission. He did not display any clinical features suggesting alcohol withdrawal. A medical workup, including a urine toxicology screen, was unremarkable.

On admission, he reported worsening depression, suicidal ideation, and auditory hallucinations of his deceased parents asking him to join them by killing himself. He was irritable, antagonistic, and difficult to redirect. He terminated the assessment and left as he wanted to be left alone. He was started on mirtazapine 15 daily and haloperidol 10 mg daily for agitation and psychosis. Mirtazapine was increased to 30 mg daily on day three to address depression, insomnia, and anxiety. However, symptoms of depressed mood, irritability, anger, antagonism, and withdrawal worsened. On day four, he demonstrated prominent perseveration and indecisiveness about treatment and medications. He declined food for three days and lost over 7 pounds over a week of hospitalization. On day ten, he was given lorazepam 1 mg for agitation and irritability; within a few hours of receiving lorazepam, his mood, socialization, psychomotor symptoms, appetite, delusions, perseveration, and indecisiveness. ECT was presented as an option, but he declined this treatment. Mirtazapine was discontinued due to concern about worsening symptoms with antidepressants. He was discharged home on lorazepam 1 mg twice daily and haloperidol 2.5 mg daily on day 15.

He stopped his medications one week following discharge and developed an immediate recurrence of the previous depressive and psychotic symptoms characterized by irritability, agitation, poor sleep, poor appetite, and delusions. He subsequently overdosed on a handful of medication and was found unconscious in his apartment. He was admitted to the medical floor, stabilized, and resumed on lorazepam, where his symptoms of severe depression, psychomotor symptoms, and psychosis improved with lorazepam 3 mg daily. Several trials of several antidepressants, such as mirtazapine, fluoxetine, and trazodone, resulted in immediate worsening of mood, psychosis, agitation, withdrawal, thought perseveration, indecisiveness, and suicidal ideation which improved with discontinuation of the antidepressant and starting lorazepam. Due to the multiple trials, failure of several antidepressants, and the improvement upon immediate discontinuation, he finally agreed to receive ECT. He received eight bilateral treatments of ECT in the inpatient setting, and his symptoms of depression improved. The lorazepam dose was reduced to 0.5 mg twice daily, and he was discharged with an outpatient follow-up plan. However, his symptoms of severe depression, delusions, and paranoia recurred again one week following the discontinuation of ECT while being maintained on the lower dose of lorazepam. This required rehospitalization for the third time within six months.

ECT was discussed in his third hospitalization due to a lack of support for transportation for outpatient ECT. He was trialed on olanzapine, and the dose was increased to 7.5 mg daily and lorazepam 1 mg twice daily by day three. However, his symptoms worsened on day five due to the inadvertent delay of lorazepam for 2 hours, and this resulted in significant withdrawal, antagonism, thought perseveration, paranoia, psychomotor agitation with poor self-care, and poor appetite. His symptoms continued for three days until lorazepam 2 mg was given intramuscularly on day eight, and his symptoms entirely resolved within one hour after the injection.

Olanzapine was discontinued, and he was trialed on lurasidone on day eight due to a previous good response. The dose was gradually increased to 60 mg daily with lorazepam 1 mg twice daily by day ten. Attempts to reduce lorazepam below 0.5 mg twice daily resulted in the recurrence of symptoms. Due to concern about stability on lorazepam, zolpidem was considered, as suggested by the role of zolpidem in the treatment of catatonia and the overlap between his psychomotor symptoms of depression and catatonia. Zolpidem was started on day 15 at a dose of 10 mg at night and gradually increased to 5 mg in the morning and 10 mg in the evening by day 17. He showed significant improvement and stability in his mood, psychomotor symptoms, delusions, thought perseveration, and indecisiveness. Both lorazepam and lurasidone were stopped by day 20. He was discharged on zolpidem 5 mg in the morning and 10 mg at night on day 27 with outpatient follow-up and remained stable and free of symptoms when the team followed up with him one month following discharge.

### 3.4 Case 4

A 69-year-old female with bipolar disorder I and a past medical history of hypertension and chronic obstructive pulmonary disease (COPD) was previously maintained on valproic acid 500 mg twice daily, and trazodone 100 mg at night was prescribed duloxetine 30 mg daily for chronic back pain about three weeks before admission. Within one week of starting duloxetine, she developed worsening depression, irritability, paranoia, psychomotor agitation, insomnia, and reduced appetite, with subsequent 15-pound weight loss over three weeks. She was reported to be irritable, tense, argumentative, critical, and threatening, and endorsed delusions that people were spying on her and trying to lock her husband in the hospital.

She was irritable, angry, suspicious, guarded, and intense on admission. She demonstrated occasional thought blocking. She perseverated over her medication dosage changes between home and the hospital in a repeated manner despite multiple attempts at explanation. Her valproic acid level on admission was 40 mcg/ml. Duloxetine was discontinued on day two, and she was started on thiothixene 2 mg daily based on her previous response; the dose was increased to 2 mg twice daily. As she remained irritable, valproic acid was increased to 500 mg in the morning and 750 mg in the evening, and lorazepam 0.5 mg twice daily was started on day four. Following the first dose of lorazepam, she showed immediate and significant improvement in mood, thought blocking, thought perseveration, sleep, delusions, and appetite. The valproic acid level reached 68 mcg/m on day seven. Lorazepam was discontinued on day 9, and this was followed by an immediate worsening of depression, irritability, anger, poor frustration tolerance, paranoia, sleep, thought blocking, and perseveration. No symptoms suggested benzodiazepine withdrawal. ECT was considered, and lorazepam was resumed. All the previously reported symptoms resolved the next day.

On day 12, she requested to be taken off lorazepam because she was concerned about its addictive nature, and hence, lorazepam was discontinued. This was followed by immediate worsening of mood with psychomotor agitation, thought blocking, and thought perseveration. Lorazepam was resumed again, and her symptoms resolved the next day. ECT was recommended again, but she preferred to continue lorazepam with a plan to taper off and stop following a period of stability in the outpatient setting. A few days later, she was discharged on valproic acid 500 mg in the morning and 750 mg in the evening, thiothixene 2 mg twice daily, and lorazepam 0.5 mg twice daily.

### 3.5 Case 5

A 57-year-old female with no significant medical history and a psychiatric history of bipolar I disorder presented to the hospital for worsening depression and prominent psychomotor and vegetative symptoms. She was previously maintained on a combination of lithium, olanzapine, and mirtazapine. She had multiple lengthy inpatient hospitalizations for worsening depression, social isolation, significant weight loss, failure to thrive, and inability to care for herself. She had trials of multiple groups of psychotropic medications with minimal responses and frequent hospitalizations.

On admission, she presented with depressed mood, social withdrawal, anhedonia, lack of energy and motivation, negativism, poor appetite, significant weight loss (BMI was 18 on admission), and perseveration of being left alone with refusal and prominent indecisiveness about any medication adjustments. With repeated encouragement, she resumed her previous medications (lithium 900 mg daily with subsequent levels of 0.8 mmol/L, olanzapine 20 mg daily, and mirtazapine 30 mg daily), which produced no changes in her symptoms after two weeks. She was initially withdrawn, provided limited and brief answers, disengaged with the team, and her appetite was poor. She declined ECT recommendations and reluctantly agreed only to medications.

There was a concern for subsyndromal catatonia based on severe withdrawal and negativism. However, she did not display any other catatonic features. She was started on lorazepam 0.5 mg twice daily, and within 24 hours, she showed marked improvement in her mood, appetite, socialization, and reasoning. Subsequently, she gained 7 pounds in one week. She was later discharged on lorazepam and the above medications with outpatient follow-up. She was not adherent to treatment following discharge and had multiple similar hospitalizations, but they were shorter, with complete resolution of symptoms following the treatment with lorazepam.

### 3.6 Case 6

A 75-year-old female with a history of bipolar I disorder was admitted to the hospital for worsening depression and suicidal ideation in the context of recent stressors. Her medical history is significant for hypertension, chronic kidney disease, hypothyroidism, and pulmonary embolism maintained on warfarin. She was also maintained on bupropion SR 100 mg twice daily and quetiapine 50 mg at night for her mood symptoms.

On admission, she presented with persistent and pervasive low mood, anhedonia, poor motivation, extreme anxiety, restlessness, catastrophic thinking, preservation, forgetfulness, inattention, confusion, hopelessness, insomnia, and poor appetite with a 7-pound weight loss over the week preceding her hospitalization.

Quetiapine was increased to 200 mg, bupropion was increased to 150 mg twice daily, and mirtazapine was added and titrated to 45 mg daily by day four. However, the previous symptoms worsened, and she demonstrated significant indecisiveness about taking medications. She later became paranoid and suspicious about the staff and her roommate and repeatedly asked the team for their credentials. Risperidone was briefly trialed, and the dose was increased to 2 mg daily with no benefit by day 10. ECT was considered, but she was unable to consent to treatment due to indecisiveness. Lorazepam 0.5 daily was added on day 12, and there was a notable improvement in anxiety, psychomotor agitation, and ability to understand and make decisions—all of which allowed her to consent to ECT.

ECT was started on day 15, and lorazepam was discontinued before treatment. Following the first bilateral ECT treatment, she showed marked improvement in her mood, anxiety, and paranoia. However, these symptoms recurred again the next day. She refused her second ECT treatment, and the team decided to pursue an ECT court order. While waiting for an ECT court order, lorazepam 0.25 mg twice daily was started on day 18 for the treatment of anxiety. Within 24 hours, her mood, anxiety, paranoia, insomnia, confusion, and indecisiveness improved. Hence, ECT was resumed on day 20, and lorazepam was discontinued before the second treatment. However, her mood worsened with significant withdrawal, isolation, extreme anxiety, poor sleep, and indecisiveness on day 21. Lorazepam 0.25 mg twice daily was resumed, resulting in substantial improvement of mood, paranoia, anxiety, insomnia, appetite, and indecisiveness on day 22. She received four additional ECT treatments, and lorazepam was stopped after the sixth treatment, with no recurrence of symptoms of depression. She was discharged after seven inpatient ECT treatments with a plan to continue outpatient ECT. However, she declined further treatment and was lost to follow-up.

### 3.7 Summary of cases

In the above cases, four patients had a primary diagnosis of bipolar disorder, and two patients had a primary diagnosis of major depressive disorder. Four patients had worsening depression and psychotic symptoms with antidepressants, while the remaining two had no benefit from antidepressants. ECT was considered in all patients. However, only three patients agreed to ECT. In patients treated with ECT, lorazepam alleviated indecisiveness and allowed consent for treatment.

Except for the 1^st^ case, none of the patients met the DSM-5-TR criteria for catatonia. However, if the prominent psychomotor symptoms and perseveration, which are commonly seen in severe depression, are also counted as features of catatonia, a significant overlap with catatonia can be considered in all these cases. Nevertheless, in the 1^st^ case, catatonia was an initial presentation that resolved with lorazepam. At the same time, depressive symptoms, psychomotor symptoms, and psychosis persisted after the resolution of catatonia and improved with the use of a higher dose of lorazepam. In the other cases, lorazepam not only immediately improved psychomotor symptoms and perseveration but also treated severely depressed mood, intense anxiety, irritability, indecisiveness, paranoia, delusions, poor appetite, and sleep within 24 hours of administering lorazepam. These symptoms recurred when lorazepam was discontinued and improved immediately upon resumption, demonstrating a clear on-and-off pattern across the patient group.

**Table 3** provides a summary of the clinical features of our cases.

**Table 3:** Summary of clinical features of our cases.

Features of Melancholia	Case 1	Case 2	Case 3	Case 4	Case 5	Case 6
MDD	X	X				
Bipolar disorder			X	X	X	X
Profoundly depressed mood	X	X	X	X	X	X
Severe anhedonia	X	X	X	X	X	X
Disabling anxiety	X	X	X	X		X
Irritability	X		X	X	X	X
Psychomotor agitation	X		X	X		X
Psychomotor retardation		X	X	X	X	
Significant weight loss	X	X	X	X	X	X
Poor sleep	X	X	X	X		X
Thought perseveration	X	X	X	X	X	X
Indecisiveness	X	X	X	X	X	X
Excessive guilt	X	X				
Delusions	X	X	X	X		X
Cognitive slowness				X		X
Response to antidepressants	No response. Received before hospitalization	Worsening depression and psychosis	Worsening depression and psychosis	Worsening depression and psychosis	No change	Worsening depression and psychosis
Days to show response on lorazepam	1 day	1 day	1 day	1 day	1 day	1 day
The final dose of lorazepam required for recovery	2.5 mg twice daily	0.5 mg twice daily	1^st^ admission: 3 mg daily On the last admission: Zolpidem 15 mg daily	Lorazepam 0.5 mg twice daily	Lorazepam 0.5 mg twice daily	Lorazepam 0.25 mg twice daily
Worsening upon lorazepam discontinuation	X	X	X	X	X	X
ECT treatment	Yes	No	Yes	No	No	Yes
Final treatment	ECT	Lorazepam	Zolpidem	Lorazepam	Lorazepam	ECT

## 4 Discussion

Our case series described six patients who presented with symptoms of severe depression and melancholic features. Patients with melancholia are known to have an increased risk for suicide due to the severity of symptoms, presence of irritability, agitation, poor sleep, and, as discussed earlier, possible worsening of symptoms with modalities commonly used for treatment, such as antidepressants (25). In our case series, all patients either had an inadequate response or worsening of their symptoms of depression, psychomotor changes, and psychosis with the use of antidepressants. This population might represent a significant portion of treatment-resistant depression (TRD) cases (32). While such results might be exciting and promising, these results should be carefully analyzed due to the small sample, the possible unique phenotype of depression, and the potential antidepressant effect of lorazepam.

The recommended treatment for severe depression with melancholic features includes ECT (31). Limited access to ECT and the legal barriers related to consent in cases of incapacity from severe depression can create further treatment challenges (33, 34). This makes the presence of an alternative pharmacotherapy of great importance, even if adjunctive. The use of lorazepam in some of our patients who were recommended for ECT resulted in improvement of their symptoms of psychomotor retardation and vegetative symptoms and restoration of their capacity; this allowed them to consent for ECT, minimized delay in treatment, and enhanced patient autonomy.

Several attempts have been made to address the ambiguity, lack of specificity, and predictive value in the diagnostic criteria for melancholia for the development of alternative constructs (32). Based on the historical review and our cases, we propose provisional [Mahgoub] criteria for the recognition of melancholia in patients with major depressive disorder or bipolar disorder who present with a depressed episode and have the following criteria: prominent psychomotor agitation or retardation or an alternating state of excitation and retardation, vegetative symptoms, perseveration/rumination, and indecisiveness (**Table 3**). We believe this better represents the historical concept of melancholia and its link to treatment. Delusions were seen in 5/6 patients; hence, they were not included in the criteria.

The significant overlap in nosology and historical background and the remarkable response of our patient’s mood, cognitive, and psychomotor symptoms to lorazepam and other GABA agonists drive further exploration of other areas of overlap, mainly phenomenology and treatment response of mood symptoms with various benzodiazepines. This can be summarized in the following sections:

### 4.1 Phenomenological overlap between catatonia and melancholia

Phenomenologically, Kahlbaum recognized the extent of the overlap between catatonia and melancholia, suggesting that melancholia can progress to catatonia and appear during the transition between melancholia and mania (3, 4).

Taylor reported that some patients with melancholia can present with profound psychomotor retardation and become stuporous (35). He added that they might barely respond to stimuli, stare into space, and sit or lay in bed for hours. He described that such a state might be striking in those without observable depressive features—such as depressed mood, hopelessness, and suicidal feelings—as these severe psychomotor symptoms might mask them. He also suggested that these patients typically stop eating and drinking. While his description of the above symptoms occurred in the context of melancholia, he interchangeably related these features of severe psychomotor retardation of stupor, withdrawal, posturing, mutism, and staring to be related to catatonia. For these patients, he suggested the use of intravenous sodium amobarbital or intramuscular lorazepam, which may abolish the stuporous or catatonic features and then reveal the typical depressive thoughts and feelings. He also suggested that patients with melancholia can have ruminations and anxiety, which can suggest an obsessive-compulsive disorder. Taylor suggested that depressive ruminations and perseverative motor behaviors associated with melancholia are usually associated with vegetative signs, which are not seen in OCD (35).

Perseveration is an additional symptom that occurs in both depression and catatonia, and it was described in four of the six patients we reported. Perseveration is defined differently between depression and catatonia. In the context of depression, perseverative thinking is a term used to describe processes such as worry and rumination, in which individuals experience repetitive, prolonged, and recurrent negative thoughts about themselves, their symptoms, their problems, or their concerns (36). Perseveration or rumination is seen in severe depression, especially with intense anxiety features, and it correlates with suicidality. In the context of catatonia, it is defined as repeatedly returning to the same topic or persisting with the same movements (14).

As Taylor and Fink provided their previously described model for melancholia, they described psychomotor agitation or restlessness as a feature of melancholia (31). In addition, Koukopoulos et al. described the clinical features of “melancholia agitata” or agitated melancholia (25). They described a state of internal tension, restlessness, irritability, unprovoked aggression, lack of self-control, and other features of mental excitement and thought ruminations. Such motor and cognitive excitement features are also seen among patients with catatonia presenting with variable manifestations of excitement, agitation, impulsivity, and compulsive-like speech described in catatonia (2, 14).

Another example of phenomenological overlap between depression and catatonia appears in pathological indecisiveness, described in depression, and ambitendency, described in catatonia. Pathological indecisiveness was observed in all 6 of the cases we reported. Pathological indecisiveness, or aboulomania, was described by Heinroth to be a cardinal feature of depression where there is a diminished will (37). Indecisiveness was later associated with severe depression and anxiety (38). Ambitendency, a symptom of catatonia, is defined as motor indecisiveness or being stuck (14). Ambivalence or inability to make decisions was described as a feature of catatonia (2). Both pathological indecisiveness described in depression and catatonic ambitendency or ambivalence described in catatonia appear to be related phenomena, with the ambitendency focusing on motor activities. While most of the symptoms in Mahgoub’s criteria were discussed by other authors (1, 35), indecisiveness, which was present in all our reported patients, was not previously reported by other authors in the context of melancholia.

### 4.2 Overlap in treatment between melancholia and catatonia

Although modern pharmacotherapy for depression has primarily focused on serotonergic and noradrenergic modulation consistent with the monoamine theory (39), numerous researchers have examined the utility of GABAergic agents for the treatment of depression. Studies conducted between the 1960s and the mid-1990s reviewed the role of several benzodiazepines in treating depression (40–58). The results of these studies were variable, and factors such as heterogeneity of study design and variations in diagnostic criteria preclude unequivocal conclusions concerning their efficacy in depression. The diagnostic criteria in these studies, influenced predominantly by the DSM-3 and DSM-3-R, have been criticized for the lack of specificity and lack of representation of core features of melancholia. Multiple agents within the benzodiazepine class with divergent pharmacokinetic and pharmacodynamic properties were studied. In addition, study settings, sample size, and different pharmacokinetics and pharmacodynamic properties of other groups of benzodiazepines were discussed.

Overall, these studies showed significant antidepressant activities for some benzodiazepines (adinazolam, alprazolam, and lorazepam) that were equal to or sometimes better than parallel comparisons of tricyclic antidepressants. These antidepressant properties were noted to be weak or absent with other benzodiazepines such as diazepam and chlordiazepoxide. Despite the antidepressant properties, no remarkable difference was observed between melancholic and non-melancholic subtypes of depression, as it was defined at that time (50, 58).

The response to treatment of agitated depression might provide a clearer view of the overlap of treatment between depression with melancholic features and catatonia. Kouropoulos et al. highlighted that these patients might worsen with antidepressants, and they respond to ECT or benzodiazepines, which are the options commonly used for the treatment of catatonia (25, 59, 60). Kahn et al. described the role of benzodiazepines in the treatment of agitated depression in a case series of three patients with agitated depression (61). All three patients had poor or unfavorable responses to trials of TCA, multiple benzodiazepines, antipsychotics, and ECT “in the first case.” Interestingly, they all demonstrated complete remission of their presenting symptoms with alprazolam and recurrence when alprazolam was discontinued in the first case, which resolved within three days after resuming alprazolam.

**Table 4** summarizes demographic and clinical features and responses to alprazolam from cases [Kahn et al. (61)].

**Table 4:** Summary of demographic and clinical features of [Kahn et al. (61)] case series on agitated depression and response to alprazolam.

Features of Melancholia	Case 1	Case 2	Case 3
Age/sex	61/F	77/F	32/F
MDD	X	X	X
Profound low mood	X	X	X
Disabling anxiety	X	X	X
Psychomotor agitation	X	X	X
Anorexia	X	X	X
Significant Weight loss	X	Not reported	Not reported
Poor sleep	X	X	X
Delusions	–	–	X
Perseverations	–	X	–
Response to benzodiazepines other than alprazolam	Did not respond to lorazepam, diazepam, and triazolam	–	–
Days to show response on alprazolam	1	3	4
The dose of alprazolam required to show improvement	0.25 mg Thrice daily that was further titrated to 1 mg Thrice daily	0.25 mg Thrice daily	6 mg daily, was further increased to 9 mg daily
Worsening upon alprazolam discontinuation	Not reported	Present and remitted in 3 days of restarting alprazolam	Not reported
ECT treatment	Ineffective	Not tried	Not tried
Final treatment	Nortriptyline 25 mg Thrice daily in addition to alprazolam 1 mg Thrice daily	Alprazolam 0.25 mg Thrice daily	Alprazolam 1 mg Four times daily and Imipramine 225 mg daily

M, Male; F, Female.

## 5 Conclusion

The present study argues for a closer examination of the relationship between melancholia and catatonia based on a case series and historical review. The cases also support a revised diagnostic concept of melancholia in patients with depressive episodes (**Table 5**). Our paper reviews the significant overlap between catatonia and depression with melancholic features in their phenomenology, the development of their nosology, and their response to treatment. A substantial body of literature has established the role of GABA agonists in the treatment of depression, including the use of several benzodiazepines.

**Table 5:** Provisional Mahgoub criteria for melancholia.

1. Psychomotor agitation	Speech: rapid, increased volume, and loud Motor: increased activity, rapid, repetitive, restlessness, and aggression
2. Psychomotor retardation	Speech: Slow, low volume, reduced production of speech. Motor: Reduced activity, isolation, withdrawal, poor eye contact
3. Prominent vegetative symptoms	Disrupted/dysregulated oral intake, sleep, decreased energy, or fatigue
4. Perseveration or rumination	Perseveration is a thought process disorder manifested by repetition or fixation on topics or themes, regardless of accuracy. The focus is on the process of fixation, not the idea or theme. Literature used both perseveration and rumination interchangeably, with an occasional focus on the content rather than the process in the case of rumination.
5. Pathological Indecisiveness (aboulomania)	Profound difficulty or inability to make sustained choices such as in treatment or life decisions.

To meet the criteria, a patient with a depressive episode should present with either criteria 1 or 2 or an alternation of 1 and 2 in addition to criteria 3,4 and 5.

We propose provisional [Mahgoub] criteria for patients with severe depression and anhedonia who also have prominent psychomotor agitation or retardation or an alternating state of excitation and retardation, vegetative symptoms, perseveration/rumination, and indecisiveness to represent better the historical concept of melancholia and its link to treatment. The overlap between melancholia and catatonia might suggest that both presentations are part of the same spectrum. The role of GABA agonists, such as lorazepam, should be explored, as it might be an option for some patients with treatment-resistant depression with these suggested [Mahgoub] criteria for melancholia, especially with the striking on-and-off effect observed in our cases.

Future development of standardized assessment tools for melancholia, based on our proposed Mahogub’s criteria and their use in prospective studies, can provide better guidance, improvement, and validation for melancholia criteria and their correlation with response to lorazepam or other GABA-agonists.

## 6 Limitations

While the result of our study that shows a clear on-and-off confirmation of lorazepam’s effect supports its efficacy for treating melancholia, the small size of the sample results in a limited ability to generalize these results. Finally, the absence of a standardized instrument for the assessment of psychopathology and severity of specific symptoms is another limitation.

## Data availability statement

The original contributions presented in the study are included in the article/supplementary material. Further inquiries can be directed to the corresponding author.

## Ethics statement

The studies involving humans were approved by Penn State IRB. Copy for IRB statement attached. The studies were conducted in accordance with the local legislation and institutional requirements. Written informed consent for participation was not required from the participants or the participants’ legal guardians/next of kin in accordance with the national legislation and institutional requirements. Written informed consent was obtained from the individual(s) for the publication of any potentially identifiable images or data included in this article.

## Author contributions

YM: Writing – original draft, Project administration, Methodology, Conceptualization. AP: Writing – review & editing. DH: Writing – review & editing. HG: Writing – review & editing, Project administration, Methodology. SN: Writing – review & editing. CM: Writing – review & editing. AF: Writing – review & editing, Supervision.
